# Venetoclax Penetrates the Blood Brain Barrier: A Pharmacokinetic Analysis in Pediatric Leukemia Patients

**DOI:** 10.7150/jca.81795

**Published:** 2023-04-25

**Authors:** Mohamed Badawi, Rajeev Menon, Andrew E. Place, Tammy Palenski, Gauri Sunkersett, Richard Arrendale, Rong Deng, Sara M. Federico, Todd M. Cooper, Ahmed Hamed Salem

**Affiliations:** 1 AbbVie, Inc, North Chicago, IL, USA.; 2 Dana-Farber/Boston Children's Cancer & Blood Disorders Center, Boston, MA, USA.; 3 Genentech, Inc, South San Francisco, CA, USA.; 4 Department of Oncology, St. Jude Children's Research Hospital, Memphis, TN, USA.; 5 Cancer and Blood Disorders Center, Seattle Children's Hospital, Seattle, Washington, USA.; 6 Clinical Pharmacy, Ain Shams University, Cairo, Egypt.

**Keywords:** venetoclax, pharmacokinetics, blood-brain barrier, cerebrospinal fluid, central nervous system.

## Abstract

Infiltration of malignant cells into the central nervous system in hematological malignancies correlates with poor clinical outcomes. Investigations into the penetration of venetoclax into the central nervous system have been limited. We report venetoclax pharmacokinetics in plasma and cerebrospinal fluid samples from a Phase 1 study in pediatric patients with relapsed or refractory malignancies that demonstrate venetoclax ability to cross into the central nervous system. Venetoclax was detected in cerebrospinal fluid (CSF) samples, with concentrations ranging from < 0.1 to 26 ng/mL (mean, 3.6 ng/mL) and a plasma:CSF ratio ranging from 44 to 1559 (mean, 385). Plasma:CSF ratios were comparable among patients with AML and ALL and no clear trend was observed in the ratios over the course of treatment. Moreover, improvement in central nervous system (CNS) involvement status was observed in patients who had measurable concentrations of venetoclax in the CSF. CNS resolution was observed for up to six months while on treatment. These findings highlight the potential role of venetoclax and provide the opportunity to further investigate its utility in improving clinical outcomes for patients with CNS complications.

## Introduction

Central nervous system (CNS) involvement, where malignant cells infiltrate the CNS, meninges, or cerebrospinal fluid (CSF), is a complication that occurs across hematological malignancies, and correlates with high-risk features and poor clinical outcomes 1. The incidence of CNS involvement varies depending on the type of malignancy. In pediatric acute myeloid leukemia (AML) and acute lymphoblastic leukemia (ALL) the incidence is 5% and 10% respectively 2, 3. These rates increase in the relapse setting 4. Management of CNS involvement includes cranial radiation, intrathecal chemotherapy and/or high-dose systemic chemotherapy 3, 5, 6.

Venetoclax is a first-in-class, potent, oral B-cell lymphoma (BCL-2) inhibitor that is approved for treatment of chronic lymphocytic leukemia (CLL) and adult AML 7 and is currently under development for several other hematological malignancies including ALL, multiple myeloma (MM), mantle cell lymphoma (MCL) and pediatric AML 8. While venetoclax plasma pharmacokinetics (PK) is well characterized, insights regarding venetoclax penetration into the CNS are limited.

To penetrate into the CNS, venetoclax would need to overcome physicochemical and physiological barriers. Venetoclax is a “large, small molecule drug” (molecular weight of 868.44), which is hypothesized to limit passage through the blood-brain barrier's (BBB) tight junctions. Moreover, venetoclax is a substrate of several efflux transporters such as P- glycoprotein (P-gp) and breast cancer resistance protein (BCRP) that are expressed within the BBB and can actively pump venetoclax outside the CNS 9. Given the high potency of venetoclax in inducing apoptosis in BCL-2-dependent malignancies, achieving even limited exposure within the CNS may still prove useful in managing CNS involvement in hematological malignancies. In this communication, we report the passage of venetoclax into the CNS by analyzing PK samples from a phase 1 study (NCT03236857) 10 that evaluated the use of venetoclax in combination with chemotherapy in pediatric patients with relapsed and refractory acute leukemia.

## Materials and Methods

The study reported here (NCT03236857) was conducted in accordance with Good Clinical Practice Guidelines and the ethical principles that have their origin in the Declaration of Helsinki. The protocol was approved by the institutional review board at each study site and each patient provided written informed consent before any study-related procedures were performed. Participants were < 25 years of age (with preference to pediatric patients < 18 years) with relapsed or refractory malignancies, had adequate hepatic and renal function. Additional details on inclusion and exclusion criteria are reported elsewhere 11.

Pediatric patients received daily doses of 400 mg or 800 mg (adult equivalent dose) of venetoclax. Doses were adjusted upon concurrent administration of moderate or strong CYP3A4 inhibitors to achieve comparable venetoclax exposures. Chemotherapy was administered based on the type of malignancy as specified in the study protocol (azacytidine, decitabine or cytarabine for AML patients; dexamethasone and/or vincristine and/or peg-asparaginase OR cytarabine and/or etoposide and/or peg-asparaginase for ALL patients). Intrathecal chemotherapy was allowed during the screening period and when clinically indicated throughout the study. Intrathecal therapy included cytarabine and hydrocortisone, with or without methotrexate. Intensive plasma PK sampling from pediatric patients was performed in Cycle 1 of treatment, followed by sparse plasma PK sampling on scheduled visits throughout the study. Venetoclax was measured in CSF as a surrogate for BBB penetration. CSF samples for PK assessments were collected at screening and while on treatment during scheduled visits if a lumbar puncture was performed as standard of care. CSF and blood PK samples were collected at the clinic. On days when a clinic visit was scheduled, patients were instructed not to administer venetoclax until they arrived at the clinic to facilitate trough PK sampling. The venetoclax concentrations in the CSF and plasma samples were determined using liquid-liquid extraction followed by liquid chromatography (LC) with tandem mass spectrometric detection (MS/MS) 12. The lower limit of quantitation for venetoclax was 2.14 ng/mL in plasma and 0.1 ng/mL in CSF. The accuracy and precision for venetoclax in both plasma and CSF were within 15%.

## Results

The analysis included samples from 46 patients with relapsed or refractory AML (n = 24), ALL (n = 18) or other hematological malignancies (n = 4). The median age of the patients was 8.5 years (range < 1 year - 25 years). Twenty (43%) of the patients were male.

**Figure 1:**
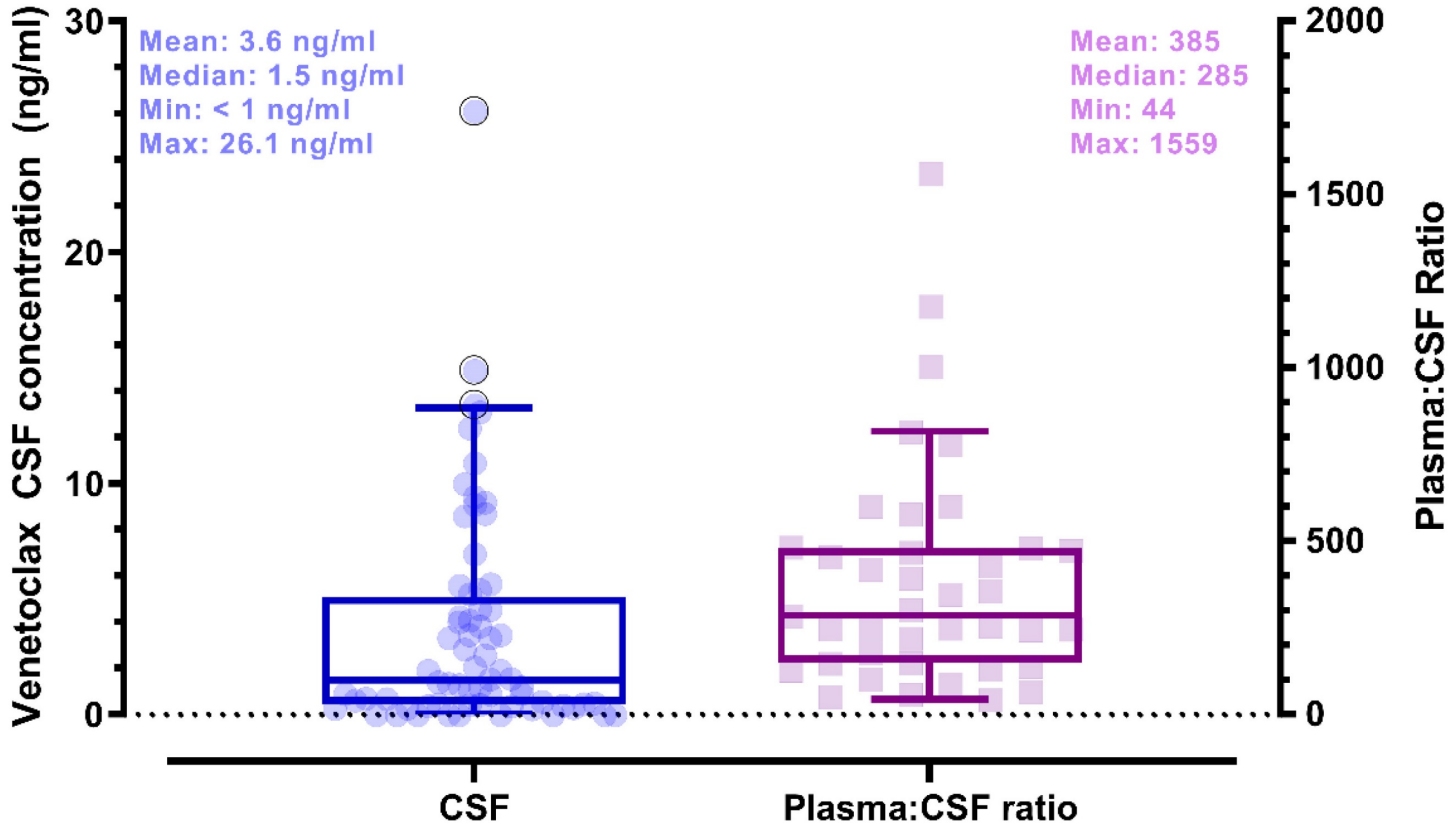
**Venetoclax Observed CSF Concentrations (left) and Plasma to CSF Ratios (right) in Pediatric Patients**. Shaded circles and squares represent observed data. Box plots show median (solid line) and interquartile range (box). Whiskers represent the 5^th^ and 95^th^ percentiles. (whiskers). Plasma:CSF ratios were calculated from CSF and plasma samples collected on the same day. Note: CSF samples below the limit of quantification were imputed to 0 for calculation of summary statistics.

**Figure 2:**
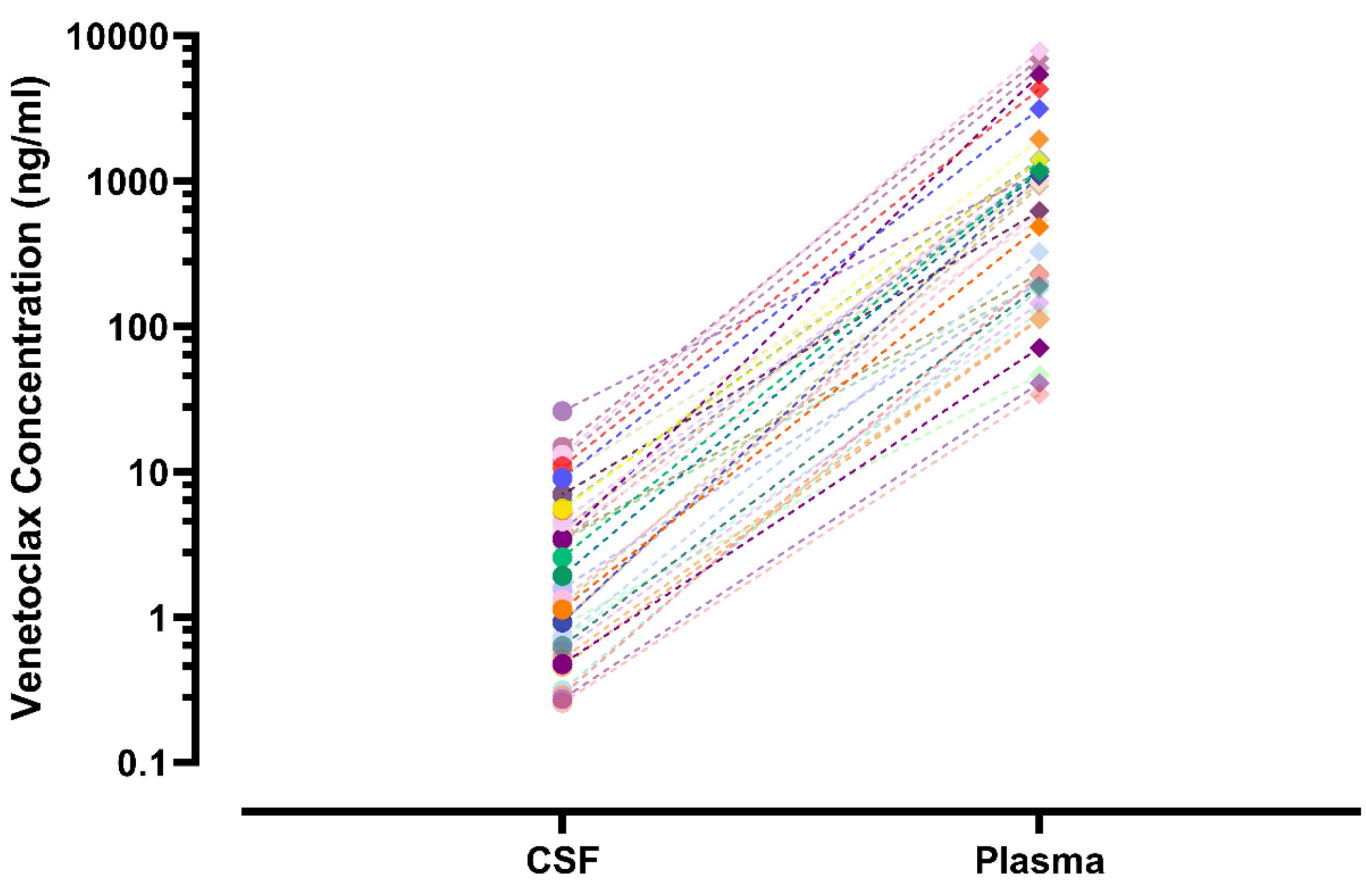
** Venetoclax CSF and Plasma Concentrations in Matched Samples from Pediatric Patients.** CSF and plasma samples were collected on the same day. Matched samples are shown as the same symbol and connected by various lines on a log scale. CSF, cerebrospinal fluid

**Figure 3:**
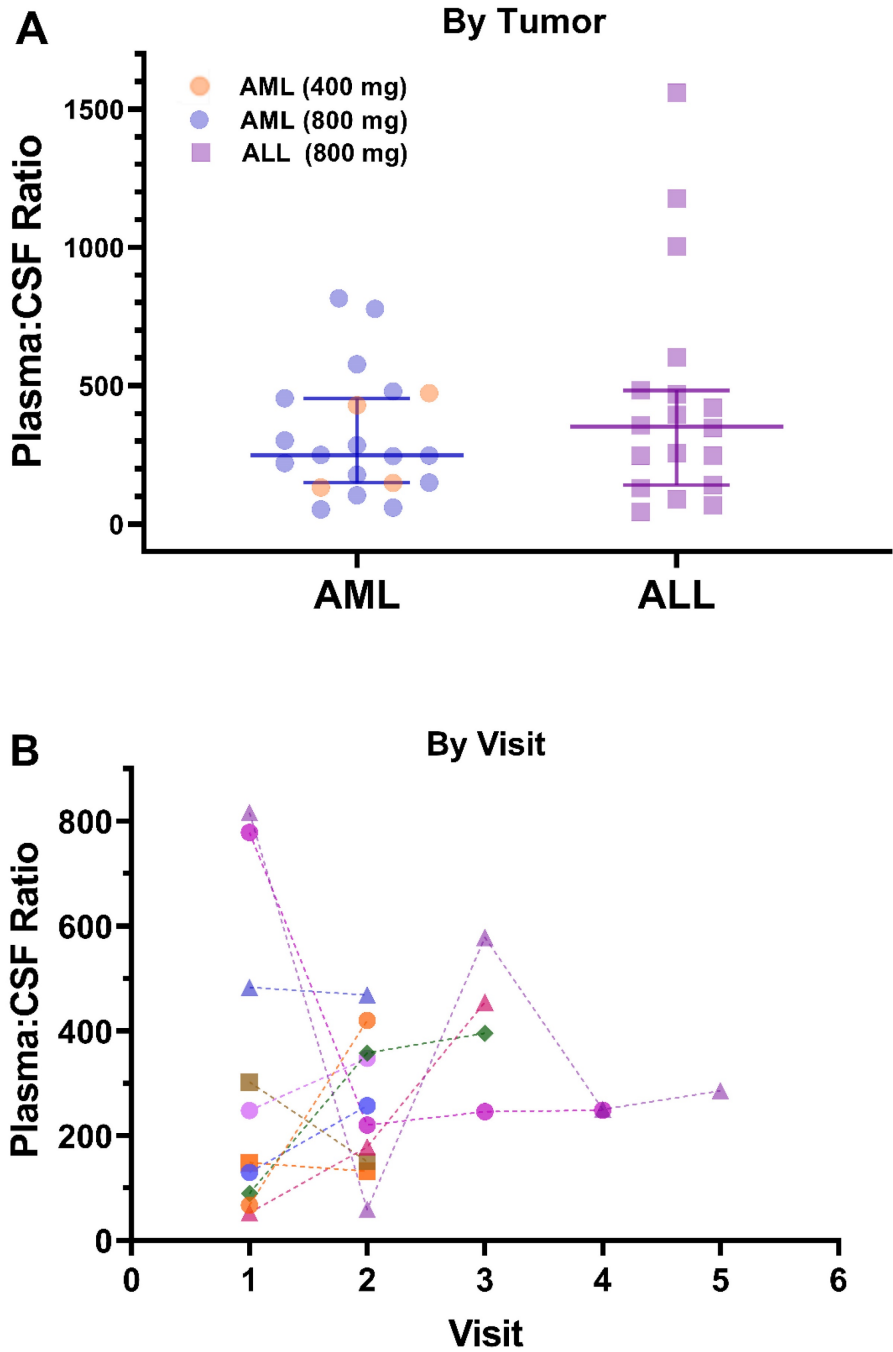
** Venetoclax Observed Plasma:CSF Ratios by A) Tumor Type and Dose Level and B) Visit.** Plasma:CSF ratios were calculated from CSF and plasma samples collected on the same day. Each shape/color combination represents data from an individual patient in Figure 3B. ALL, acute lymphoblastic leukemia; AML, acute myeloid leukemia; CSF, cerebrospinal fluid

Plasma and CSF samples from pediatric patients receiving venetoclax at either 400 or 800 mg (adult equivalent doses) in combination with chemotherapy were evaluated. A total of 92 CSF samples were collected. Venetoclax was detected in most of the CSF samples. The mean venetoclax concentration in CSF was 3.6 ng/mL with concentrations ranging from < 0.1 to 26 ng/mL (Figure 1). To better understand accumulation of venetoclax in the CSF as it relates to systemic concentrations, the plasma:CSF ratio was calculated for the subgroup of patients who had a plasma sample collected on the same day of the lumbar puncture. Thirty-nine CSF samples, collected from 23 patients, had a plasma PK sample collected on the same day. All but two patients within this subgroup had detectable venetoclax in the CSF and plasma. The CSF concentrations observed within this subgroup (n = 23) were similar to that of the overall group (mean concentration of 4.0 ng/mL and range between < 0.1 and 26 ng/mL in the subgroup vs. 3.6 [range < 0.1 - 26 ng/mL] in the overall group). We further calculated the plasma-to-CSF (plasma:CSF) ratio to compare the CSF concentrations relative to the plasma concentrations observed on the day of sampling. Plasma concentrations ranged from < 2 ng/mL to 8 ug/mL, and the mean plasma:CSF ratio was 385 with a range of 44 - 1559 (Figure 1). Figure 2 presents the matched venetoclax CSF and plasma concentrations for patients within this subgroup. Generally, higher CSF concentrations were observed with higher plasma concentrations. Within this subgroup, most patients had received an 800 mg dose (or adult equivalent dose) of venetoclax while four patients received venetoclax at 400 mg. While the number of patients receiving 400 mg was limited, no apparent trend was observed in CSF accumulation between the two dose levels (Figure 3). Moreover, plasma:CSF ratios were comparable among patients with AML and ALL (Figure 3) suggesting that penetration into the CNS is unlikely to be affected by the tumor type.

Nine patients (4 ALL patients and 5 AML patients) out of the 46 patients included in the analysis demonstrated CNS involvement either at screening (n = 5) or after initiation of therapy (n = 4). Of those, seven patients had subsequent CSF assessments after initial detection of CNS involvement. All seven patients demonstrated resolution of CNS involvement at their subsequent assessments while on treatment. The venetoclax CSF concentrations in these patients (n = 7) ranged from 0.30 to 7.7 ng/mL (mean 2.7 ng/mL). The remainder two patients who demonstrated CNS involvement did not have further CSF assessments after initial detection and hence it could not be determined if the CNS status improved or not. Noteworthy, for patients with more than one calculated plasma:CSF ratio, no clear trend was observed in the ratios over the course of treatment (Figure 3).

## Discussion

CNS involvement is a poor prognostic factor in hematological malignancies whose management requires aggressive therapy including cranial radiation, intrathecal chemotherapy, or high dose systemic chemotherapy. Venetoclax is a first in class BCL-2 inhibitor which has demonstrated marked efficacy in CLL and AML either as monotherapy or in combination, in the frontline or relapsed/refractory setting. In this study, we aimed to evaluate the ability of venetoclax to cross the BBB and its potential utility in management of CNS involvement. To our knowledge, venetoclax CSF concentrations were only reported in individual case reports, and a detailed CSF analysis in a broad population as presented herein has not been reported.

Venetoclax is highly protein bound with less than 1% free unbound drug. The mean calculated plasma:CSF ratio was 385, comparable to previously reported ratios 13, 14 and reflective of the limited free drug that is available to distribute into tissues, in line with high protein binding. This ratio, however, is more than 4-fold higher than the blood-to-brain ratio observed preclinically in mice (data on file at AbbVie). This lower venetoclax disposition into brain observed in humans is contrary to our expectation, given the higher expression of P-gp in mice BBB compared to humans 9. While concentrations observed in the CSF may not be fully indicative of disposition within the brain tissue, it provides reasonable evidence of the ability of venetoclax to penetrate the BBB and suggests that other anatomical and/or physiological factors are involved in disposition of venetoclax into the CNS.

Venetoclax is a potent BCL-2 inhibitor and is extremely effective in inducing cell death in AML and ALL as demonstrated in the literature. In AML, the median half maximal inhibitory concentration (IC50) of treatment of venetoclax in multiple primary AML specimens treated ex-vivo was well below 10 nM (8.7 ng/ml) 15. Similar results were demonstrated in ALL, where primary ALL cells were highly sensitive to venetoclax with five out of the six samples tested having an IC50 < 10 nM 16. In our analysis, the mean venetoclax CSF concentration observed was around 4 ng/ml. While the mean observed CSF concentration is lower than 10 nM, it is important to note that, unlike ex-vivo models where drug exposure is transient (48 h in AML and 96 h in ALL), in the clinic, patients are chronically dosed and leukemia cells are under continuous exposure to venetoclax. This supports the assumption that *in-vivo* concentrations lower than *ex-vivo* IC50s could drive clinical benefit to patients with AML and ALL having CNS involvement. This is demonstrated by improvements in CNS status within seven patients in this study where the mean venetoclax CSF concentration was 2.7 ng/ml with improvement in CNS status maintained for up to 6 months in an ALL patient and up to 5 months in an AML patient. Similar observations were reported in case reports in patients with AML and CLL. Reda et al. and Beziat et al. in patients with CLL 13, 14, 17. Reda et al. was first to report venetoclax concentrations close to the IC50 of CLL cells in the CSF of a CLL patient (1. 5 - 2.8 ng/ml) receiving 400 mg of venetoclax 13. Moreover, Beziat et al. demonstrated that administering venetoclax in combination with systemic chemotherapy was tolerated and resulted in well-controlled CNS manifestations in another CLL patient with minimal side effects 17. In fact, neurologic improvements were maintained under continuous venetoclax exposure, six months after the last methotrexate infusion, with absence of new symptoms 17. Most recently, venetoclax CSF concentrations ranging 4 - 7 ng/ml were reported in an AML patient with leptomeningeal involvement where the patient received continuous venetoclax which was associated with significant improvement in muscle tone 14. While the therapeutic effect on CNS involvement observed in our analysis could partly be attributed to the intrathecal therapy received by patients (cytarabine and hydrocortisone, with or without methotrexate), the role of venetoclax in contributing to and/or enhancing the effect is certainly to be considered given its high potency and effectiveness in hematological malignancies. Venetoclax, as a monotherapy or in combination, has consistently achieved robust response rates in frontline or relapsed/refractory CLL 18-20. Moreover, in AML and ALL, venetoclax has demonstrated high complete remission rates, 66.4% and 59.6%, respectively 21, 22. Collectively, these findings and clinical evidence highlight the potential use of venetoclax as a well-tolerated medication to control and maintain CNS localizations in hematological malignancies.

In conclusion, these findings demonstrate the ability of venetoclax to penetrate into the CNS. This distribution into the CNS, coupled with its potent apoptotic effects suggest clinical utility in management of CNS complications in hematological malignancies.

## Abbreviations

ALL: acute lymphoblastic leukemia
AML: acute myeloid leukemia
BBB: blood-brain barrier
BCRP: breast cancer resistance protein
CLL: chronic lymphocytic leukemia
CNS: central nervous system
CSF: cerebrospinal fluid
LC: Liquid chromatography
MCL: mantle cell lymphoma
MM: multiple myeloma
P-gp: P- glycoprotein
PK: pharmacokinetics
